# NMOSD IgG Impact Retinal Cells in Murine Retinal Explants

**DOI:** 10.3390/cimb45090463

**Published:** 2023-09-07

**Authors:** Hannah Nora Wolf, Veronika Ehinger, Larissa Guempelein, Pratiti Banerjee, Tania Kuempfel, Joachim Havla, Diana Pauly

**Affiliations:** 1 Department of Experimental Ophthalmology, University Marburg, 35037 Marburg, Germany; 2 Institute of Clinical Neuroimmunology, Biomedical Center and University Hospital, Ludwig-Maximilians-Universität München, 81377 Munich, Germany

**Keywords:** NMOSD, autoantibodies, retina, chemokine, complement, local, Müller cell, mouse retinal explants

## Abstract

Neuromyelitis optica spectrum disorders (NMOSD) are chronic inflammatory diseases of the central nervous system, characterized by autoantibodies against aquaporin-4. The symptoms primarily involve severe optic neuritis and longitudinally extensive transverse myelitis. Although the disease progression is typically relapse-dependent, recent studies revealed retinal neuroaxonal degeneration unrelated to relapse activity, potentially due to anti-aquaporin-4-positive antibodies interacting with retinal glial cells such as Müller cells. In this exploratory study, we analysed the response of mouse retinal explants to NMOSD immunoglobulins (IgG). Mouse retinal explants were treated with purified IgG from patient or control sera for one and three days. We characterized tissue response patterns through morphological changes, chemokine secretion, and complement expression. Mouse retinal explants exhibited a basic proinflammatory response ex vivo, modified by IgG addition. NMOSD IgG, unlike control IgG, increased gliosis and decreased chemokine release (CCL2, CCL3, CCL4, and CXCL-10). Complement component expression by retinal cells remained unaltered by either IgG fraction. We conclude that human NMOSD IgG can possibly bind in the mouse retina, altering the local cellular environment. This intraretinal stress may contribute to retinal degeneration independent of relapse activity in NMOSD, suggesting a primary retinopathy.

## 1. Introduction

Neuromyelitis optica spectrum disorders (NMOSD) represent a rare subset of autoimmune diseases that affect the central nervous system (CNS). The primary areas of pathological involvement are the optic nerve and spinal cord. NMOSD is distinct from multiple sclerosis due to the presence of autoantibodies against the astrocytic water channel protein aquaporin-4 (AQP4-IgG) [1]. Binding of autoantibodies to AQP4 initiates the classical complement pathway, leading to inflammation and downstream neurodegeneration. The course is relapsing in 90% of cases, and chronic progressive courses without relapse-dependent worsening are rare. Interestingly, in AQP4-IgG positive NMOSD patients, a loss of retinal ganglion cells and inner plexiform layer (GCIP) volume has been observed, independent of optic neuritis attacks [2,3,4,5,6,7]. Hence, an underlying primary retinopathy may be considered as an explanation in the context of NMOSD.

AQP4 is primarily found in astrocytes and glial cells in the CNS as well as in the retina [8,9,10]. In AQP4-IgG positive NMOSD, primary retinopathy may be suspected due to the interaction of AQP4-IgG with retinal Müller cells or astrocytes. Müller cells maintain retinal homeostasis by regulating the fluid and ion homeostasis, neurotransmitter recycling, secretion of growth factors, and inflammatory mediators [11,12,13,14]. AQP4-IgG have been shown to decrease the expression of AQP4 on Müller cells, disrupting volume regulation and inhibiting cell proliferation [15]. This mechanism may contribute to retinal degeneration with or without inciting optic neuritis.

Classically, the primary sites of inflammation and lesion formation in NMOSD were believed to be the optic nerve and spinal cord, with retinal thinning considered as a secondary consequence. However, recent studies have demonstrated that AQP4-IgG can bind to both human and rodent retinas in vivo and ex vivo [16,17,18], resulting in retinal degeneration independent of optic nerve inflammation, suggesting the possibility of a primary retinopathy [2,4,5,19,20].

In this exploratory study, we examine the putative impact of NMOSD IgG on retinal chemokine homeostasis. Chemokines are inflammation regulators involved in various biological processes, such as wound repair and cell morphogenesis. They attract leukocytes and stimulate both endothelial and epithelial cells. In particular, chemokines CCL2 and CCL5 have been found to be increased in response to AQP4-IgG treatment in isolated rat astrocyte cultures [21]. However, the implications for CXCL2 and CXCL10 remain ambiguous, as divergent findings have been reported in the serum of NMOSD patients across various studies [22,23,24,25]. Although chemokines CCL3 and CCL4 are known to be elevated in the serum of NMOSD patients, their concentrations within the retinal milieu are yet to be explored [26,27].

Alongside chemokines, complement proteins play a crucial role as inflammatory mediators in NMOSD. Blocking the complement system has been proven effective in preventing disease relapses in AQP4-IgG-positive NMOSD [28]. However, the connection between complement activation, retinal neuroaxonal degeneration, and inflammation still remains unknown. Furthermore, the prospective effect of therapeutic intervention in retinal tissue remains uncertain, as the prevailing treatments are delivered systemically [29,30].

The retina is typically safeguarded from systemic inflammatory mediators by the blood–retinal barrier, which dichotomically also bars most therapeutic molecules from entering. Consequently, it is important to identify tissue-specific reactions to comprehend the possible impact of NMOSD IgG within the retinal compartment and potentially guide the development of more targeted treatments. 

Previous studies have been conducted using various in vivo models, such as rodents, and primary astrocyte cultures [17,18,21,31,32]. In vivo models provide the benefit of examining the impact of external factors, such as human antibodies, in their entirety, encompassing the effects of systemic blood circulation. However, single cell cultures may not represent the full spectrum of cellular effects, as they exclude cell–cell and cell-systemic interaction. Retinal explants offer the advantage of studying retinal cell interactions with a stressor independent of blood. It is crucial, though, to recognize that the ex vivo environment might not supply optimal nutrients to the retina, leading to a baseline level of cellular stress. Therefore, it is essential to compare the effects of NMOSD IgG with control IgG to obtain reliable results.

The goal of this pilot study was to explore a probable primary retinopathy and the potential impact of NMOSD IgG on the retina. Here, we focused specifically on retinal cellular responses without considering the involvement of the systemic immune system, blood-born hormones or growth factors. We examined the release of inflammatory mediators, such as chemokines, and the expression of local complement system transcripts. In an ex vivo mouse retinal explant model, we demonstrated that retinal cells possibly respond to NMOSD IgG binding with an acute, anti-inflammatory chemokine response. These findings might provide new insights into the role of NMOSD IgG in retinal inflammation and the potential for targeted therapeutic interventions. In conclusion, understanding the chronic effects of AQP4-IgG on the retina could help unravel the mechanisms behind relapse-independent progressive retinal degeneration of the GCIP.

## 2. Materials and Methods

### 2.1. Cultivation and Treatment of Mouse Retinas

Female *C57BL6/J* mice aged 6 to 12 weeks were used in this study and were housed in a 12 h light/dark cycle with an illumination level of approximately 400 lux. Female mice were chosen as the exclusive subjects in this study due to the higher prevalence of NMOSD in females compared to males, ensuring a more representative sample for the investigation. All experiments were conducted in accordance with the European Community Council Directive 2010/63/EU and the ARVO Statement for the Use of Animals in Ophthalmic and Vision Research. CO_2_ inhalation was used to sacrifice the mice and their eyes were immediately enucleated and immersed in Dulbecco’s phosphate buffered saline (PBS, #D8537, Thermo Fisher Scientific, Dreieich, Germany) supplemented with 100 mg/mL streptomycin and 100 µg/mL penicillin (#15140122, Thermo Fisher Scientific, Waltham, MA, USA).

The cornea, lens, and vitreous were removed and the optic nerve was transected near the eyeball. The neural retina was carefully isolated from the underlying sclera and retinal pigment epithelium and each retina was cut in half, resulting in two retinal segments per retina and four retinal explants per animal. One segment was treated with purified control IgG and one with NMOSD IgG to compensate for preparatory effects. However, it should be noted that retinal explant viability and inflammatory status may vary depending on the particular preparation, which may result in high standard errors within treatment groups.

The tissue was transferred photoreceptor layer down to a 12-well cell culture insert (#10567522, Corning incorporated Life Sciences, Tewksbury, MA, USA) and cultured under serum-free conditions in Neurobasal-A medium (#10888022, Thermo Fisher Scientific, Waltham, MA, USA) supplemented with 2% B27 (#0080085SA, Thermo Fisher Scientific, Waltham, MA, USA), 1% N2 (#17502048, Thermo Fisher Scientific, Waltham, MA, USA), 2 mM GlutaMAX (#35050038, Thermo Fisher Scientific, Waltham, MA, USA) and 100 units/mL penicillin-100 µg/mL streptomycin. The retinas were cultivated at the air-medium interface under standard conditions (37 °C, 5% CO_2_, 80% humidity), and half of the basal medium was changed daily.

IgGs were purified using a Protein A column (#GE11-0034-94, Cytiva Lifesciences, Shrewsbury, MA, USA) to exclude the involvement of the systemic immune system, blood-born hormones or growth factors. Purified IgGs from five controls and five AQP4-IgG-seropositive NMOSD patients were pooled. All samples were obtained from the neuroimmunological biobank of the LMU Hospital, and samples as well as clinical data were provided irreversibly anonymized (protocol number 163-16, Table A1). Retina medium was supplemented with IgG at a concentration of 100 µg/mL. IgG-conditioned media was added at the start of the culture and with every medium change.

### 2.2. Hematoxylin and Eosin (HE) Staining

The morphology of untreated retinas was assessed using HE staining. Then, 20 µm thick cryosections were immersed in hematoxylin, rinsed with H_2_O_dest_, and washed with tap water. Subsequently, slides were incubated in eosin solution and dehydrated. The dehydrated slides were then immersed in xylene and covered with entellan.

### 2.3. TUNEL Staining

Retinas were fixed with 4% paraformaldehyde (#100496, Merck, Schwalbach, Germany) in PBS (pH 7.4) for 2 h and then washed in PBS. Dehydration was carried out overnight in 42% sucrose solution (#107687, Merck, Schwalbach, Germany) followed by embedding in frozen section medium NEG-50 (#11912365, Thermo Fisher Scientific, Braunschweig, Germany) and cryosectioning into 20 µm sections using a Leica CM1950 cryostat (Leica, Wetzlar, Germany). To analyse apoptotic cell death, TUNEL staining was performed using a DeadEnd Fluorometric TUNEL kit (#G3250, Promega, Fitchburg, WI, USA) following the manufacturer’s instructions.

### 2.4. Quantitative Real-Time PCR (qRT-PCR)

Retinal explants were cultured under three conditions: without treatment, with the addition of purified control IgG, or purified NMOSD IgG. After one or three days in culture, the samples were prepared for qPCR analysis. mRNA isolation was performed using a NucleoSpin RNA/Protein Kit (#740933.50, Macherey-Nagel, Düren, Germany), followed by cDNA synthesis using a Quantitect Reverse Transcription Kit (#205311, Qiagen, Hilden, Germany). 

qRT-PCR analysis was conducted using custom-designed primer sets (*c1s*: F: CCCTGTAGCCACTTCTGCAA, R: GGGCAGTGAACACATCTCCA, *c3*: F: AGCCCAACACCAGCTACATC, R: GAATGCCCCAAGTTCTTCGC, *cfh*: F: AAAAACCAAAGTGCCGAGAC, R: GGAGGTGATGTCTCCATTGTC, *aqp4:* F: AGCAATTGGATTTTCCGTTG, R: TGAGCTCCACATCAGCACAG, *idh3b*: F: GCTGCGGCATCTCAATCT, R: CCATGTCTCGAGTCCGTACC), and the Rotor Gene Sybr green PCR Kit (#204076, Qiagen, Hilden, Germany). The *idh3b* transcript was used as a housekeeping gene for normalization. Additionally, IgG-treated retinal expression data were normalized to untreated retinal expression data. 

### 2.5. Bead-Based Multiplex Chemokine/Cytokine Immunoassay and Human AQP4-IgG ELISA

Chemokine and cytokine protein concentrations in culture supernatants were assessed after three days of cultivation using a customized mouse ProcartaPlex Multiplex Immunoassay (#EPX010-20440-901, Invitrogen AG, Carlsbad, CA, USA). The assay was conducted following the manufacturer’s protocol and analysis was performed on a MAGPIX™ instrument (Luminex Corporation, Austin, TX, USA).

To determine AQP4-IgG titer of control sera, NMOSD patient sera, and purified IgG samples, an AQP4-IgG sandwich ELISA kit (#RAQP4/96/2R, BioVendor GmbH, Kassel, Germany) was utilized. The ELISA was performed in accordance with the manufacturer’s instructions, and the absorbance was measured at a wavelength of 450 nm on a VarioScan Flash ELISA-reader (Thermo Fisher Scientific, Waltham, MA, USA).

### 2.6. Coomassie Staining

IgG purification success was verified through protein separation of serum and purified antibody fractions from one patient in a 12% SDS-PAGE under reduced conditions. Following separation, proteins were stained using Coomassie solution (#ISB1L-1L, Sigma-Aldrich, Missouri, USA). Visualization of the protein bands was conducted using a FluorChem FC2 Imaging System (Alpha Innotech, San Leandro, CA, USA).

### 2.7. Immunohistochemistry (IHC)

IHC was carried out as previously described [33]. Retinas were washed with PBS, fixed in 4% paraformaldehyde (#100496, Merck, Schwalbach, Germany) for 2 h, dehydrated in 42% sucrose (#107687, Merck, Schwalbach, Germany) overnight, and embedded in NEG-50 frozen section medium (#11912365, Thermo Fisher Scientific, Braunschweig, Germany). Cryosections of 20 µm were obtained using Leica CM1950 (Leica, Wetzlar, Germany). Retinas were incubated with the primary antibody specific for GFAP (1:500, #ab7260, Abcam, Cambridge, UK) or control/patient antibodies in 0.5% Triton-X100/2% BSA/PBS at 4 °C overnight. Following a PBS wash, antibody binding was detected with secondary antibodies (1:500 goat anti-rabbit Cy3-conjugated antibody, 1:500 goat anti-human FITC-conjugated antibody) diluted in 2% BSA/PBS for 2 h. Cell nuclei were stained with DAPI (1:1000). Nonspecific binding of secondary antibodies to retinal epitopes was excluded by standard validation staining (Figure A2). Fluorescent images were captured using an apochromatic stereo microscope (Carl Zeiss, Oberkochen, Germany). Immunohistochemical signal was quantified using ImageJ software (V 1.53k, National Institutes of Health, Bethesda, MD, USA).

### 2.8. Statistics

Statistical analyses were conducted using GraphPad Prism 9.3.1 (GraphPad Software Inc., San Diego, CA, USA). All data are presented as mean ± standard deviation (SD). Detailed information about specific n-values, employed statistical analyses, and coding of significance levels can be found in each figure and its corresponding figure legend. 

## 3. Results

### 3.1. In Vitro Cultivated Retinal Mouse Explants Showed Proinflammatory Baseline Characteristics

**Figure 1:**
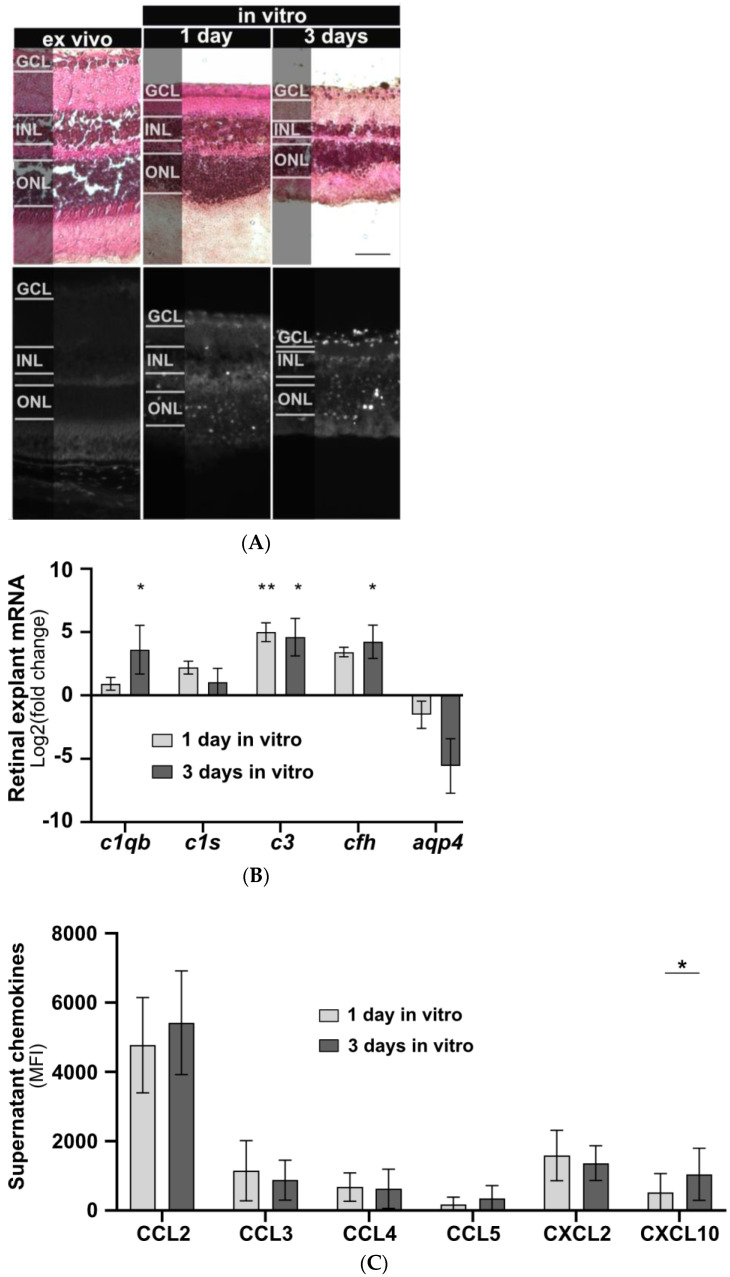
Retinal explant morphology, complement component transcription, and chemokine secretion during in vitro cultivation. (**A**) Retinal morphology of freshly fixed (ex vivo) and cultivated retinas of *C57BL/6J* mice was assessed using a HE staining to visualize cell nuclei, extracellular matrix, and cytoplasm, respectively. Retinal thinning was observed in all layers after one and three days of cultivation compared to uncultured retinas. Cell death increased during cultivation with no fluorescence detected in the ex vivo retina. Scale bar, 50 µm. (**B**) Retinal transcription of complement components changed depending on the cultivation time. *C1qb, c3*, and *cfh* mRNA expression significantly increased during cultivation. *Aqp4* mRNA decreased, and no trend was detectable for *c1s* mRNA. Bars represent mean values (n = 3) ± SD. Compared to an uncultivated control: * *p* < 0.05, ** *p* < 0.01 (ordinary one-way ANOVA, Dunnett’s multiple comparisons test). (**C**) Supernatants of cultivated retinas were collected at one and three days of untreated retinal cultivation and analysed for chemokines using a multiplex cytokine assay. CXCL10 secretion increased during in vitro cultivation. CCL2, CCL3, CCL4, CCL5, and CXCL2 secretion remained unchanged. GCL: Ganglion cell layer, INL: inner nuclear layer, ONL: outer nuclear layer. Bars represent mean values (n = 6) ± SD. * *p* < 0.05 (multiple paired *t*-tests, Holm–Sidak method).

**Figure A1:**
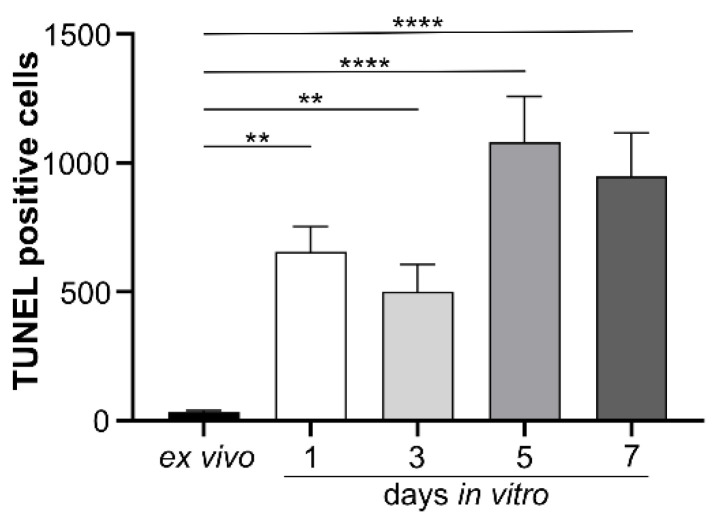
Apoptosis was assessed in all retinal layers during seven days of in vitro cultivation using the TUNEL technique. While no fluorescence was detected in the ex vivo retina, increasing apoptotic activity was observed with prolonged cultivation time. Therefore, we selected three days of retinal explant cultivation for all subsequent experiments. Bars represent mean values (n = 3) ± SD, with statistical significance denoted as ** *p* < 0.01, **** *p* < 0.0001 as determined by ordinary one-way ANOVA and Dunnett’s multiple comparisons test.

**Figure A2:**
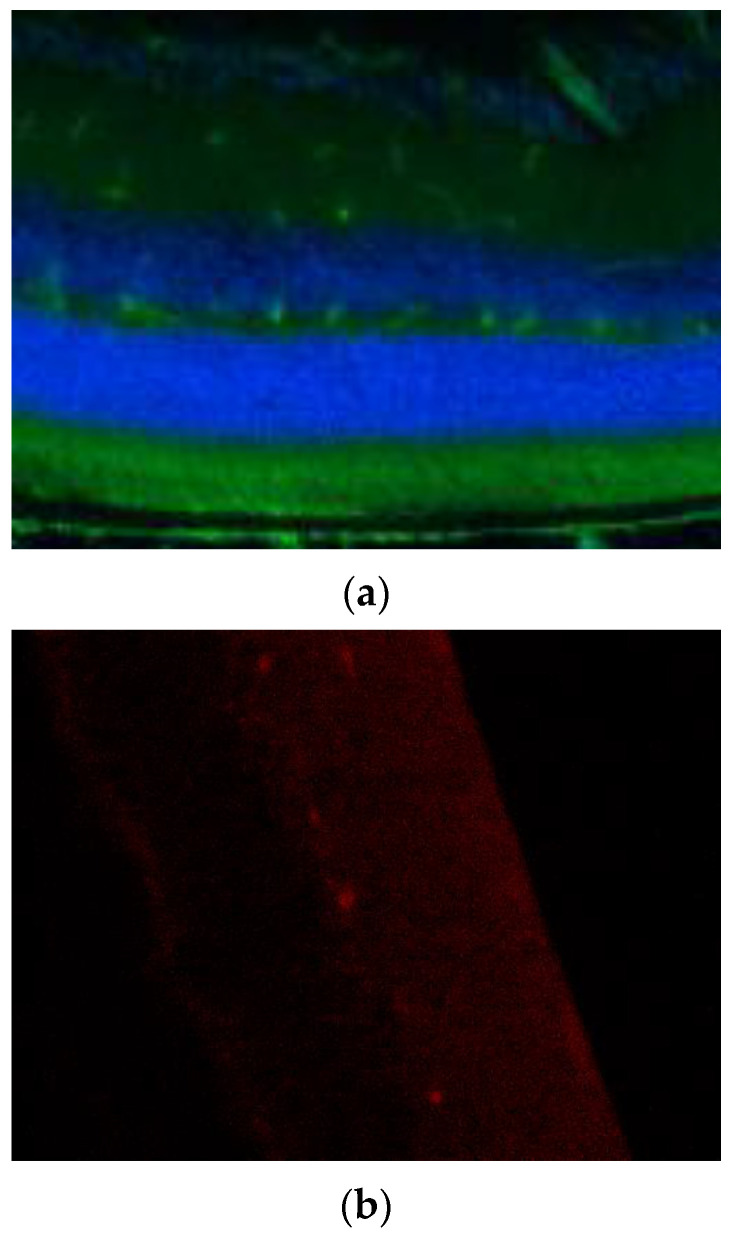
Secondary antibodies used in immunocytochemical staining were tested for unspecific binding to retinal tissue. (**a**) Negative control staining of the secondary goat–anti human FITC-conjugated antibody on uncultivated, fixated murine retinal tissue. (**b**) Negative control stainings of the secondary goat–anti rabbit Cy3-conjugated antibody on uncultivated, fixated murine retinal tissue.

The effects of isolated human IgG on mouse murine retinal cells were studied in vitro. Initially, a model for cultivating mouse retinal explants was developed, and baseline parameters for morphology, chemokine secretion, and complement component expression were determined. Retinal explants were cultivated for up to seven days in an air-medium interface on transwell filters (Figure 1). Hematoxylin and eosin (HE) staining revealed retinal thinning in all layers after one and three days of cultivation compared to an uncultured retina fixed immediately after enucleation (Figure 1A). After three days of cultivation an increase in TUNEL-positive cells in all retinal layers and a slight increase in glial fibrillary acidic protein (GFAP) staining, a marker of astrocyte and Müller cell activation, were observed (Figure 1A). In vitro cultivation of the retina for five or seven days led to significant cell death, making it unsuitable as a model system (Figure A1).

**Figure 2:**
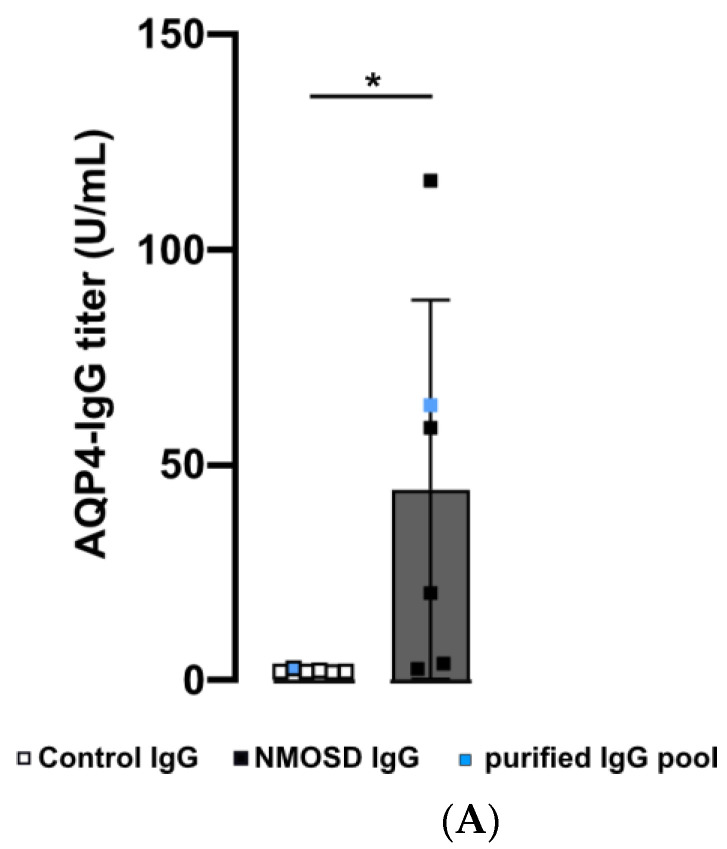

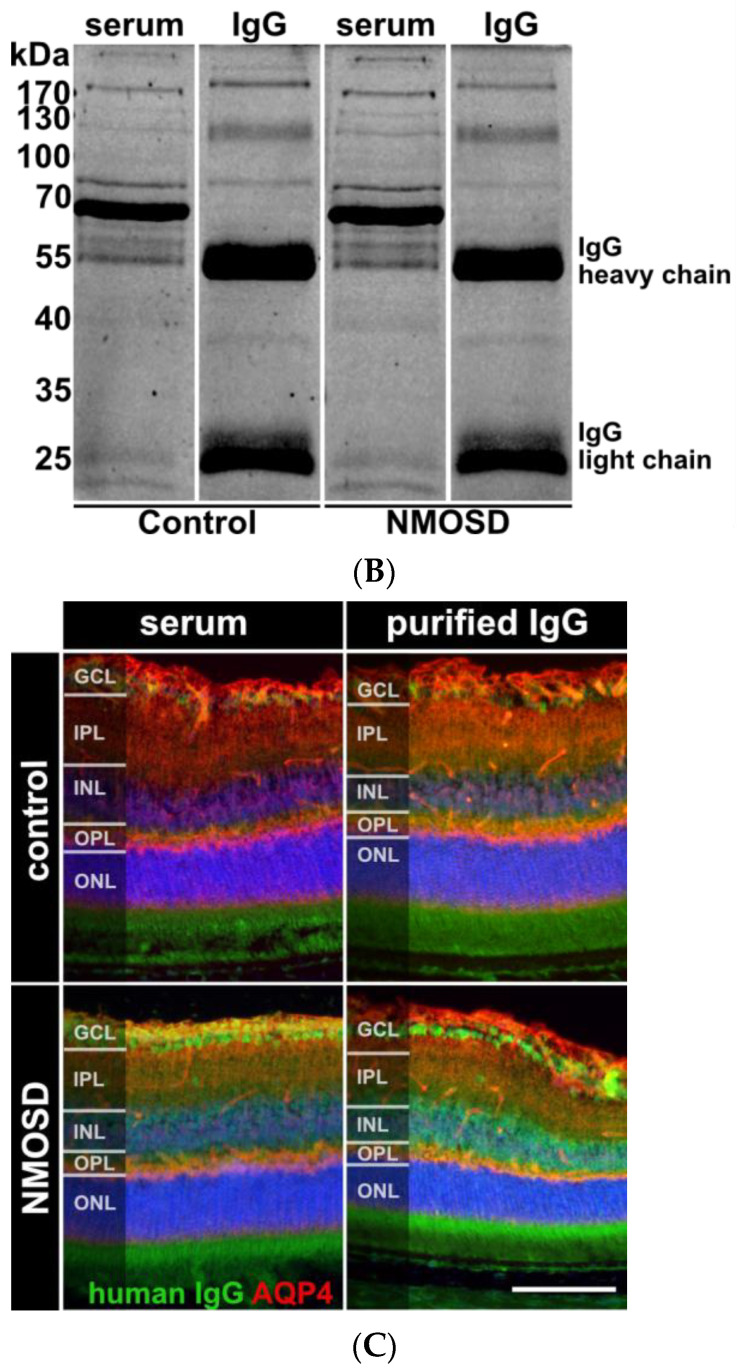
Human purified NMOSD IgG bound to the mouse retina. (**A**) AQP4-IgG reactivity was analysed in serum of controls (n = 5) and NMOSD patients (n = 5) as well as purified IgG pools (n = 1 each). Control serum samples and the control IgG pool tested negative for AQP4-IgG, while positive anti-AQP4 IgG titers were confirmed for NMOSD patient sera and the purified NMOSD IgG pool. Bars represent mean values ± SD. * *p* < 0.05. (unpaired *t*-test assuming Gaussian distribution). (**B**) The Coomassie staining of NMOSD patient serum before and after immunoaffinity purification was used to verify IgG purification. (**C**) Mouse retina sections were incubated with serum and purified IgG from controls and NMOSD patients. Human IgG binding was confirmed for serum and purified IgG from NMOSD patients, while no binding was observed for serum and IgG from controls. Scale bar, 50 μm.

AQP4, mainly expressed by astrocytes and Müller cells in the retina, is the principal cellular target for AQP4-IgG in NMOSD sera (Figure 2A). Consequently, we analysed the *aqp4* expression during cultivation of retinal explants. A decrease in *aqp4* mRNA was observed, though it did not reach statistical significance (Figure 1B).

Inflammatory processes in NMOSD are also associated with complement activation. To measure effects on the expression of classical complement pathway components, we analysed *c1qb* and *c1s* mRNA levels. After three days of cultivation, a significant increase in *c1qb* was observed (*p* = 0.034), while no change was detected for *c1s* expression (Figure 1B). To evaluate the effects on the alternative complement pathway components during cultivation, we determined *cfh* mRNA levels and found increased expression after three days of cultivation (*p* = 0.02) (Figure 1B). The central complement component *c3* showed a significant increase after one day (*p* = 0.006) and three days (*p* = 0.01) of retinal cultivation (Figure 1B).

Retinal degeneration is also associated with altered retinal protein secretion. Consequently, we observed an increase in CXCL10 secretion (*p* = 0.018) after three days of cultivation in our baseline chemokine analysis. For CCL2, CCL3, CCL4, CCL5, and CXCL2, protein secretion remained unchanged between cultivation days (Figure 1C).

In summary, mouse retinal explants revealed early signs of inflammatory activity during the initial one to three days of in vitro cultivation. This finding helps to better understand the gradual retinal degeneration process.

### 3.2. Human NMOSD IgG Bound to the Mouse Retina

In this preliminary investigation, our goal was to examine the potential influence of NMOSD IgG on retinal cells without interference of other serum components such as hormones, cytokines, chemokines, or growth factors. We isolated IgG from the serum of NMOSD patients and healthy controls. ELISA analysis verified AQP4 reactivity for both NMOSD patient sera and their respective isolated IgG pool, while neither sera nor the respective isolated IgG pool from controls showed positive results (Figure 2A). Coomassie staining of serum and isolated IgGs on an SDS gel demonstrated successful purification of IgGs from serum (Figure 2B). 

Retinal macroglia, particularly Müller cells and astrocytes, express AQP4 [8,9,34]. To investigate the interaction of human NMOSD IgG with proteins in the mouse retina, we incubated retinas fixed and cryosectioned immediately after enucleation with serum and isolated IgG fractions from NMOSD patients and controls. The binding of NMOSD serum and purified IgG to the mouse retina confirmed the reactivity of NMOSD IgG to the mouse retinal proteins. We observed immunoreactivity in the ganglion cell layer (GCL) and inner nuclear layer (INL) (Figure 2C).

### 3.3. NMOSD IgG Increased Retinal GFAP Staining, Which Is a Marker of Müller Cell Reactivity

**Figure 3:**
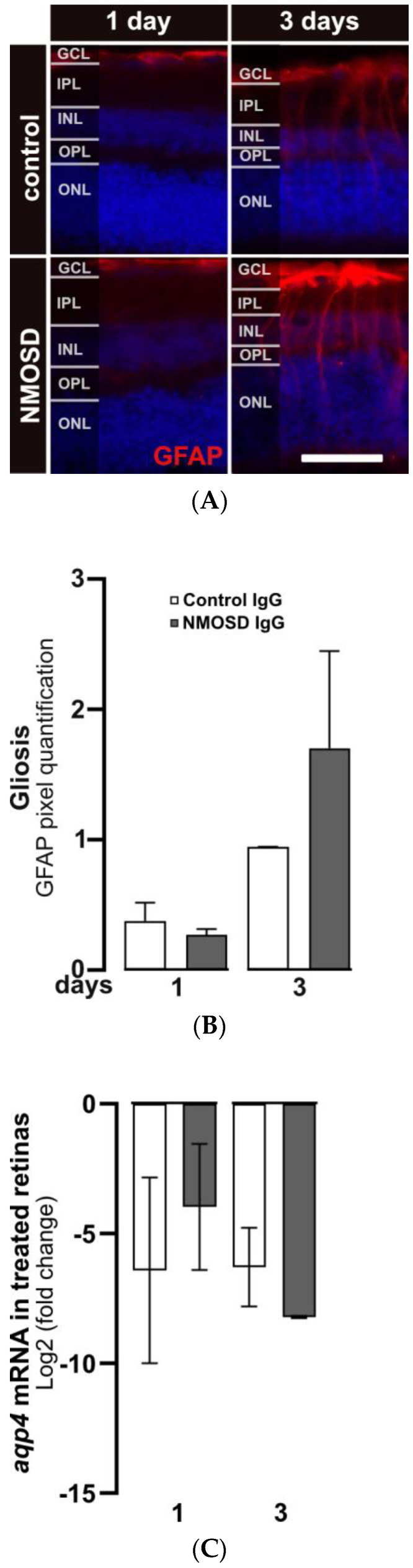
NMOSD IgG-enhanced Müller cell gliosis in retinal explant cultures. (**A**) GFAP immunoreactivity (red) was assessed by immunohistochemistry staining in retinas treated with control IgG or NMOSD IgG pool. (**B**) Quantification of GFAP immunoreactivity after one and three days of treatment with respective IgG fractions in retinal explant culture demonstrated increased GFAP levels in NMOSD IgG pool-treated retinas after three days of cultivation compared to control IgG-treated retinas. Bars represent mean values ± SD. (control—one day (n = 3); three days (n = 2); NMOSD—one day (n = 3); three days (n = 3)). (**C**) Retinal *aqp4* mRNA expression was analysed after one and three days of cultivation with control IgG or with NMOSD IgG pool (one day (n = 3), three days (n = 2)). The mRNA levels were compared to untreated, cultivated retinas. No significant changes in mRNA levels were observed due to different treatments. Bars represent mean values ± SD. (two-way ANOVA with Tukey’s multiple comparisons test).

After three days of cultivation, gliosis in response to stress in Müller cells was observed by increased staining of GFAP. GFAP protein expression in Müller cells was upregulated in both treatment groups after three days compared to one day of cultivation (Figure 3A). Quantification of GFAP signals showed a trend toward upregulated GFAP expression in NMSOD IgG-treated retinas (Figure 3B). Interestingly, transcription of the water channel protein AQP4, which is a potential target of NMOSD IgG on Müller cells, was not affected by the addition of IgG (Figure 3C).

### 3.4. NMOSD IgG Reduced the Secretion of CCL2, CCL3, CCL4 and CXCL10 in Mouse Retinal Explants

**Figure 4:**
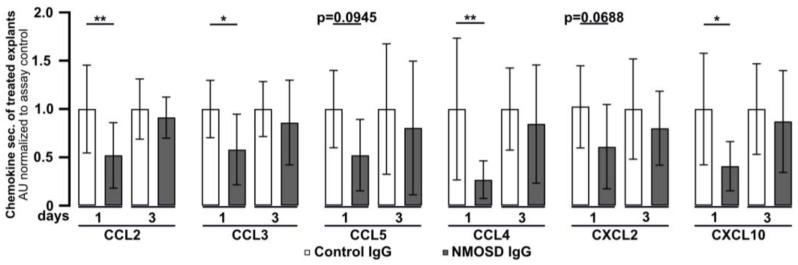
Mouse retinal explants treated with NMOSD IgG showed decreased release of CCL2, CCL3, CCL4, and CXCL10. Chemokine levels were assessed in the supernatants of control IgG or NMOSD IgG-treated cultivated retinas. CCL2, CCL3, CCL4, and CXCL10 were significantly reduced after one day of cultivation with NMOSD IgG compared to control IgG-treated retinas. Bars represent mean values (n = 10–11) ± SD. * *p* < 0.05, ** *p* < 0.01 (two-way ANOVA with Sidak’s multiple comparisons test).

Retinal explants were found to release chemokines under in vitro culture conditions (Figure 1C). To investigate the effect of NMOSD or control IgG on chemokine secretion, culture supernatants were analysed by multiplex ELISA (Figure 4). After one day of culture, secretion of the chemokines CCL2 (*p* < 0.01), CCL3 (*p* < 0.05), CCL4 (*p* < 0.01), and CXCL10 (*p* < 0.05) was significantly lower in media of explants treated with NMOSD IgG than in the supernatant of the retinas treated with control IgG. A similar trend, which did not reach significance, was observed for the chemokines CCL5 (*p* = 0.0945) and CXCL2 (*p* = 0.0688). However, after three days of treatment significant differences in chemokine secretion between the treatment groups could no longer be detected. Retinal explants were also found to secrete CCL11, IL-17A, IL-1β, IL-6, TNFα, and VEGF-A, but we did not observe differences between treatment groups.

### 3.5. NMOSD IgG Did Not Alter Complement mRNA Expression in Mouse Retinal Explants

**Figure 5:**
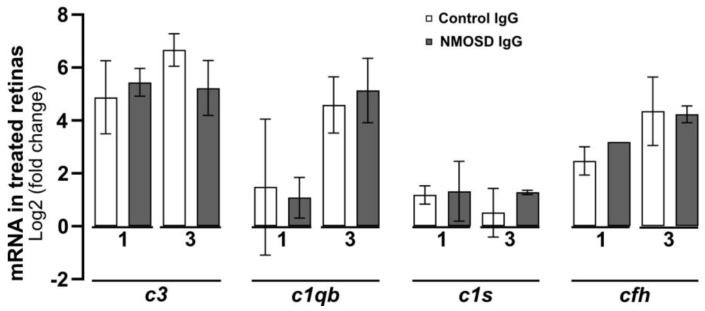
The effect of NMOSD IgG on complement mRNA expression in mouse retinal explants was investigated. The mRNA levels of complement components *c1qb, c1s, c3,* and *cfh* were analysed after one and three days of cultivation with control IgG (one day n = 4, three days n = 3) or with NMOSD IgG pool (one day n = 3, three days n = 2). The mRNA levels were compared to untreated, cultivated retinas. No significant changes in mRNA levels between the different antibody treatments were observed. Bars represent mean values ± SD (two-way ANOVA with Tukey’s multiple comparisons test).

The complement pathway is known to play a crucial role in the pathophysiology of NMOSD [35], as its activation in the fluid phase and distribution in tissues lead to inflammation, cell damage, and cell lysis [36]. In a recent study, we demonstrated that various cell types within the retina can produce complement components locally [13]. Here, we analysed the transcriptional levels of complement components in retinal explants treated with control IgG and NMOSD IgG. Our findings indicate that there were no significant changes in mRNA levels between the treatment groups for classical pathway components *c1qb* and *c1s*, alternative pathway component *cfh*, and for the central complement component *c3* (Figure 5).

## 4. Discussion

In AQP4-IgG positive NMOSD, individual relapses are often severe with poor remission rates. However, increased neurological deficits are almost always associated with relapses and not with relapse-independent progression. Recently, evidence of subclinical retinal neuroaxonal degeneration, likely due to primary retinopathy, has been demonstrated in NMOSD patients. This hypothesis is further supported by studies that have shown retinal neuroaxonal degeneration in NMOSD patients with and without a history of optic neuritis [2,4,5,19,20]. In this exploratory study, we used mouse retinal explants and treated them with purified IgG fractions from AQP4-IgG-positive NMOSD patients to evaluate their reactivity and modulatory properties against murine retinal epitopes. 

Retinal explant cultivation is a widely used model to study the pathophysiology of retinal degeneration [37,38]. In this study, we cultivated *C57BL6/J* retinas for up to seven days and examined tissue viability and inflammatory status. We observed a decrease in retinal thickness over time, and the number of TUNEL-positive cells in all retinal layers increased during cultivation, limiting the use of this model to three days. The limitation is due to the fact that the metabolic demands of a systemic blood-deprived retina exceed the amount of nutrients that can be supplied by the culture media, leading to a rapid decline in tissue viability after three days of enucleation [39,40,41]. Despite this limitation, the model offers several advantages. This includes the possibility to study primary retinopathy and its histopathologic correlates in isolation, independent of systemic facets. Furthermore, by obtaining two semi-independent retinal explants from the posterior segments of the eyes of each animal, we were able to reduce the number of animals needed for the study, promoting a more ethical use of animals in research.

Retinal neuroaxonal degeneration in AQP4-IgG-positive NMOSD is typically considered a downstream effect of inflammatory disease activity at the optic nerve and subsequent ganglion cell death [42,43]. However, controversy exists regarding whether initial retinal disease activity could serve as a second trigger for retinal degeneration in NMOSD. This is supported by rodent models that have shown NMOSD-associated antibody binding to retinal epitopes when incubated with patient-derived AQP4-IgG-positive serum or when wildtype rats are treated with AQP4-IgG [17]. These data are consistent with clinical evidence of relapse-independent retinal neuroaxonal degeneration in AQP4-IgG-positive NMOSD [2,4,5,19,20]. AQP4, expressed on the endfeet of retinal Müller cells, is a likely target for binding by AQP4-IgG in NMOSD [8,9,10,34]. Treatment with AQP4-IgG-positive serum in an immortalized Müller cell line (MIO-M1) has been shown to reduce AQP4 detection, cause AQP4 internalization, and impair Müller cell volume regulation, providing further evidence of antibody-mediated degenerative effects at the cellular level [15]. These findings provide suggestive evidence of a primary retinopathy in AQP4-IgG-seropositive NMOSD.

The secretion profiles of chemokines in inflammatory CNS diseases may offer insights into unique immunopathological processes and have potential therapeutic implications. Current studies support a critical role of chemokines in NMOSD pathogenesis. However, the regulation of chemokine levels in NMOSD patients is contradictory in the literature. For instance, while one study reported elevated levels of the systemic neutrophil-related chemokine CXCL2 in AQP4-IgG-seropositive patients compared to controls [25], another study found decreased serum CXCL2 levels [22]. Our study also found decreased local CXCL2 secretion in retinas treated with NMOSD IgG compared to control IgG. Another important chemoattractant, CXCL10, has been documented to promote T-cell adhesion to endothelial cells and facilitate blood–brain barrier disruption [44]. There is also conflicting evidence on systemic CXCL10 levels in AQP4-IgG positive NMOSD compared to patients with other neurological diseases and multiple sclerosis [23,24]. Our results demonstrated that CXCL10 secretion is reduced immediately after antibody binding and returns to normal levels after three days. In contrast, CXCL10 is upregulated in brain microvascular endothelial cells after NMOSD IgG treatment and in the optic nerve after AQP4-IgG treatment in mice [45,46]. The site-specific and time-dependent regulation of chemokine levels observed in our study may be a causal link for the possible relapse-related protein regulation seen locally in our NMOSD IgG-treated mouse retinas.

In previous studies, an upregulation of CCL2, CCL3, CCL4, and CCL5 in NMOSD patients was reported, in contrast to the local, time-dependent downregulation of chemokine secretion observed in our NMOSD IgG-treated mouse retinas [47,48,49,50]. CCL2, which is produced locally by fibroblasts, Müller cells, microglia, and astrocytes, is involved in neuroinflammation in the CNS and regulates cellular mechanics. Although CCL2 is not systemically regulated in NMOSD patients, increased local secretion is detectable in rat astrocytes after treatment with purified NMOSD IgG [21,51]. For CCL3, a microglia chemokine, an increase was reported only in the serums of one of two cohorts of NMOSD patients studied [26,27]. CCL4, on the other hand, was elevated in different cohorts of NMOSD patients [26,27]. Systemic regulation of CCL5 has not yet been reported in the literature, but local *ccl5* mRNA is upregulated in rat astrocytes after treatment with AQP4-IgG-positive serum [21]. This is consistent with the time-dependent local upregulation of CCL5 secretion in NMOSD IgG-treated mouse retinas detected after three days in this exploratory investigation.

The contradictory findings on systemic and local chemokine regulation in NMOSD suggest that modulation of proinflammatory chemokines may be tissue- and model-specific. Retinal protein concentrations may not correspond to serum concentrations [52,53,54], and the retina may attempt to protect itself from the effects of signaling molecules from the blood. Reduction in tissue-specific chemokine concentration compared to blood could be a retinal counterregulatory mechanism to attenuate local inflammatory responses. Furthermore, temporal differences may also be important to the observed discrepancies. In our study, we examined the change immediately after cell stress, whereas in vivo, the stressor is often chronically present over a long period of time, and only the late effects of the differences in chemokine levels would be visible.

AQP4-IgG, which are detectable in around 80% of NMOSD patients, lead to complement activation and associated cell death in the CNS [32,55]. Systemic inhibition of this inflammatory pathway with eculizumab, a recombinant humanized monoclonal antibody against the complement protein C5, has been shown to improve patient outcomes by preventing new relapses [28]. However, the potential contribution of intravenous C5 inhibition to minimizing primary retinopathy has not yet been studied. Our previous study showed that complement components are produced and can be activated in the retina, indicating that intravitreal C5 inhibition could potentially attenuate retinal degeneration [13,56]. However, in our current exploratory study, we did not observe modulation of local complement expression in retinal cells at a transcriptional level upon binding of human NMOSD IgG to the mouse retina, while chemokine release was regulated. It should be noted that in previous NMOSD mouse models, the human complement system always had to be injected together with passively transferred human IgG for degeneration to occur [31,32]. This also confirms that mere human antibody binding in the mouse retina is not sufficient to activate the murine complement reaction. Furthermore, passively transferred human anti-AQP4-IgG triggered lesions in the CNS of rats only if a T-cell immune response had been induced previously [57]. Nevertheless, we observed increased GFAP reactivity and altered chemokine release in retinal explants treated with NMOSD IgG, even in the absence of human complement and murine T-cell activity. Our preliminary results may support the notion that NMOSD IgG could partially cause direct cellular damage in the retina that may be independent of the complement system [17]. Despite the valuable insights obtained from this study, it is important to acknowledge the limitation of a small sample size, which calls for further comprehensive investigations to validate and expand upon our findings.

## 5. Conclusions

In this exploratory study, we aimed to investigate the potential NMOSD IgG-mediated primary retinopathy. Our findings demonstrated that IgG from NMOSD patients specifically interacted with proteins in the mouse retina, leading to increased GFAP expression in Müller cells. Moreover, treatment with NMOSD IgG resulted in diminished chemokine secretion but did not affect complement mRNA expression or cell death in the retina. These preliminary results indicate that NMOSD IgG may have an influence on retinal physiology in NMOSD, possibly through its impact on retinal cells and inflammation. This study advances our understanding of the role of NMOSD IgG in retinal pathology and highlights the importance of further therapeutic strategies for NMOSD patients.

## Data Availability

The data presented in this study are available on reasoned request from the corresponding author if the data exchange is possible with respect to valid data protection laws.

## Appendix A

**Table A1:** Demographic and clinical characteristics of patients.

NMOSD Patients	Sex	Age	AQP IgG Status
01	female	51	Positive
02	female	68	Positive
03	female	55	Positive
04	female	33	Positive
05	female	46	Positive
